# A Case of Undetermined Adenoid Cystic Carcinoma With Airway Stenosis Treated With Palliative X-Ray Radiotherapy Leading to Significant Improvement in Patient’s General Condition

**DOI:** 10.7759/cureus.36743

**Published:** 2023-03-27

**Authors:** Nobuko Utsumi, Masafumi Inoue, Kosei Miura

**Affiliations:** 1 Department of Radiation Therapy, JCHO (Japan Community Health Care Organization) Tokyo Shinjuku Medical Center, Tokyo, JPN; 2 Department of Diagnostic Pathology, JCHO (Japan Community Health Care Organization) Tokyo Shinjuku Medical Center, Tokyo, JPN

**Keywords:** respiratory distress, radiotherapy, airway stenosis, mediastinum, adenoid cystic carcinoma

## Abstract

Primary adenoid cystic carcinoma (ACC) of the upper anterior mediastinum is rarely encountered in clinical practice, and no standard treatment has been established. We performed palliative radiotherapy to improve airway narrowing in a patient with primary ACC of the mediastinum who presented with respiratory distress as their main complaint. As a result of radiotherapy, the ACC was reduced in size, the narrowed airway was opened due to compression by the ACC, and the patient's general condition improved. We present the results of this case with a review of the relevant literature.

## Introduction

Adenoid cystic carcinoma (ACC) is a type of cancer that originates in the salivary glands and accounts for 1%-2% of head and neck cancers and 10% of salivary gland tumors [1-3]. The primary treatment for locally advanced ACC is surgery, and postoperative radiotherapy is recommended [3,4], although there are no large clinical trials due to the rarity of the disease. ACC originating from outside the head and neck region, such as the larynx and trachea, is extremely rare [2,5,6]. There have been few comprehensive reports of ACC originating from areas other than the head and neck region. To the best of our knowledge, there have been no such reports from Japan, and there is no established standard treatment for this type of cancer. The present study reports on the disease course and treatment outcome of ACC in a 79-year-old woman in the upper anterior mediastinum. The purpose of this report is to offer practical knowledge for future clinical practice.

## Case presentation

**Figure 1:**
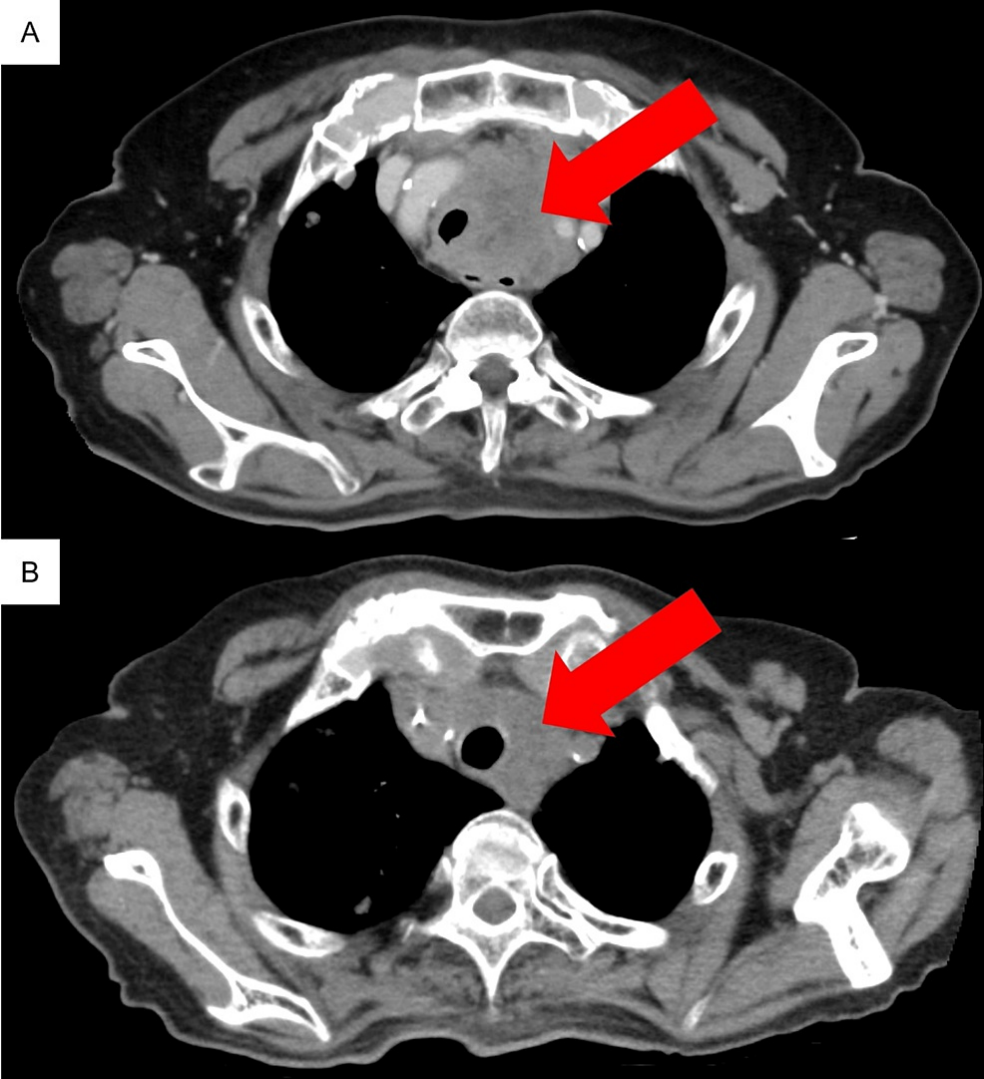
Cervical-thoracic computed tomography (CT) scans of the patient before and after radiotherapy. A) Preradiotherapy contrast-enhanced computed tomography (CT) images; B) postradiotherapy plain CT images. (A) Prior to radiotherapy, the trachea was compressed by the tumor and displaced to the right side (arrow). (B) Taken two weeks after radiotherapy, radiotherapy resulted in a marked shrinkage of the tumor and improvement in airway displacement (arrow).

**Figure 2:**
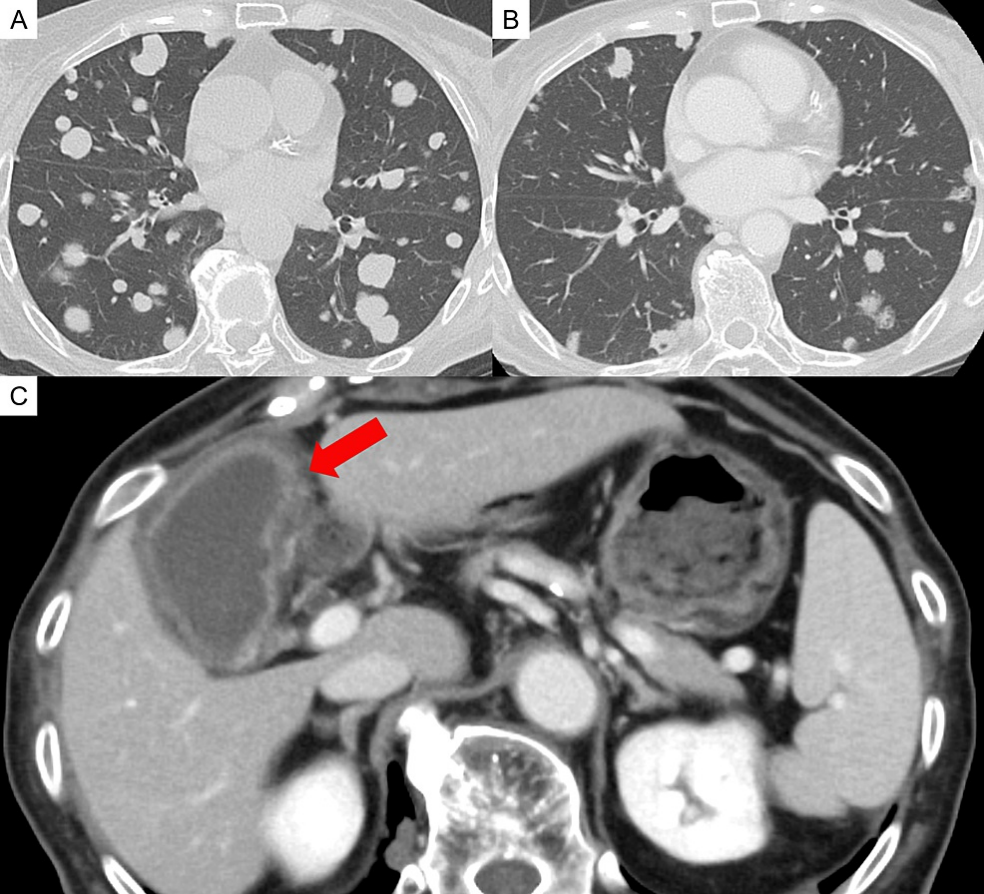
Computed tomography (CT) images at initial examination and after one month of treatment with lenvatinib. A) Computed tomography (CT) images of the lungs at the initial examination; B) CT images of the lungs after one month of treatment with lenvatinib; and C) CT images of the abdomen after one month of treatment with lenvatinib. Lenvatinib treatment reduced lung metastases (A and B) but caused edema around the gallbladder (arrow in C).

**Figure 3:**
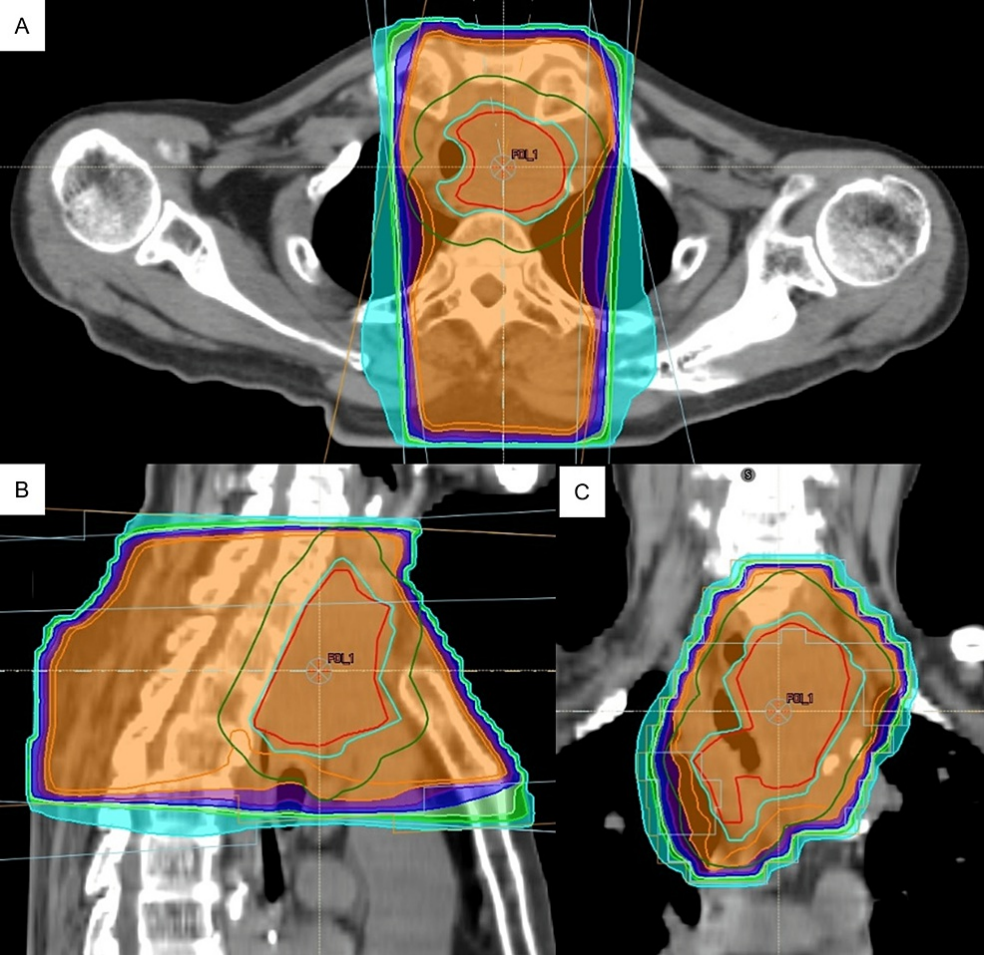
Dose distribution of radiotherapy. Representative axial (A), sagittal (B), and coronal (C) images of dose distribution are shown.

**Figure 4:**
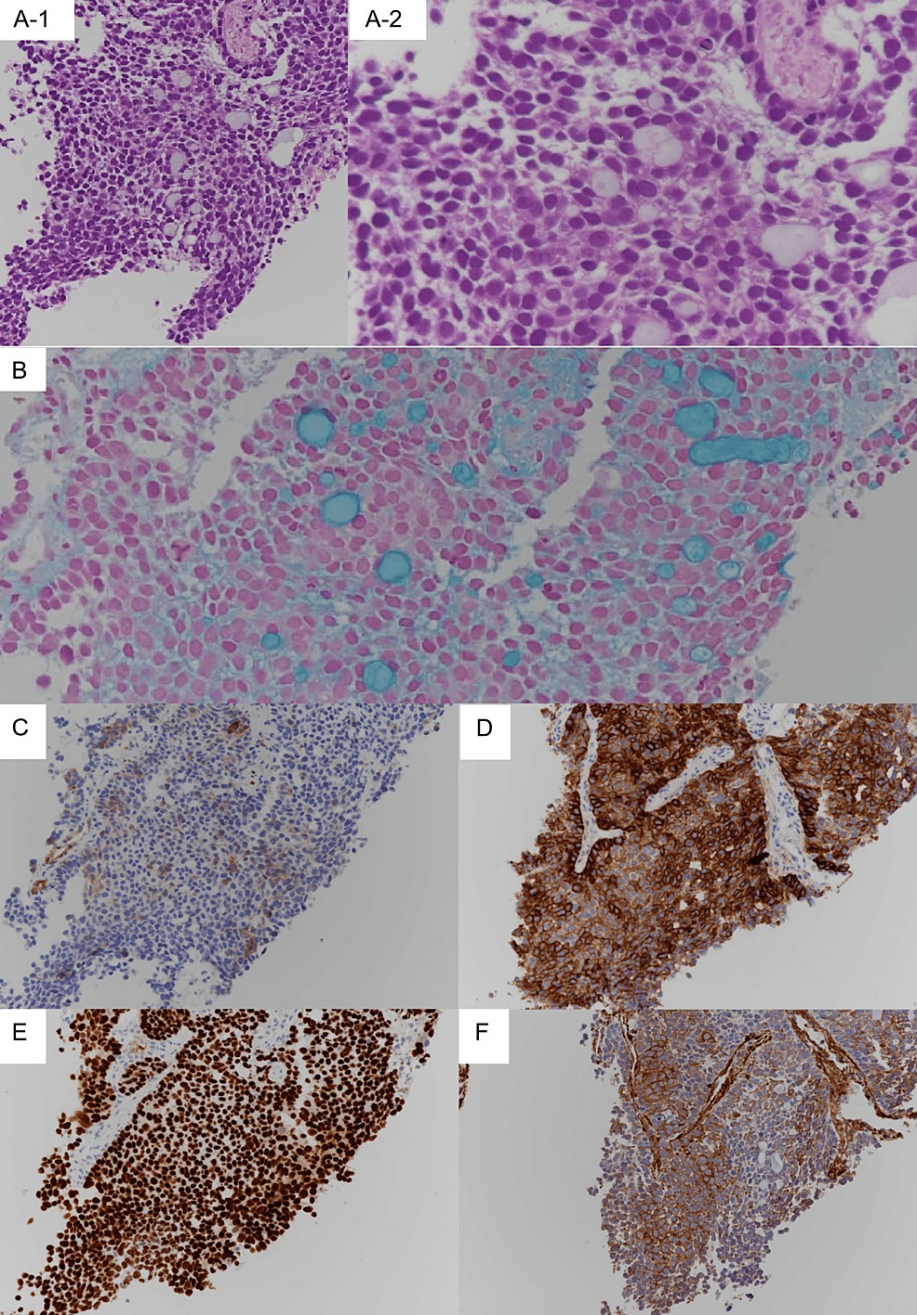
Pathological features of the tumor. (A) Hematoxylin and eosin staining indicated salivary gland-type tumor (A-1, 200x; A-2, 400x). (B) The mucoid substance was stained with Alcian blue (400x). Immunohistochemical staining images showed positive expression of (C) c-kit (weak+), (D) discovered on gastrointestinal stromal tumor-1 (+), (E) p40 (+), and (F) smooth muscle actin (+) (200x), which supported a diagnosis of adenoid cystic carcinoma.

The patient was a 79-year-old woman with a medical history of surgery for uterine fibroids and no history of malignancy. There was no significant family history to note. The patient had comorbidities including essential hypertension, dyslipidemia, and hepatic dysfunction but was only taking antihypertensive medications. She visited a local doctor on X-5 day complaining of dyspnea that started on X-10 day. She had wheezing, and a chest X-ray showed multiple nodules in both lungs. Her initial examination showed a blood oxygen saturation of 97% in room air. A contrast-enhanced computed tomography (CT) scan showed an upper anterior mediastinal tumor compressing the airway (Figure 1A) and multiple lung tumors (Figure 2A). On X-4 day, the patient was transferred to a hospital for a possible emergency tracheostomy due to severe airway stenosis, but treatment for airway stenosis was deemed difficult due to the tumor's location. The patient was transferred to our hospital on X day. Radiotherapy (30 Gy in 10 fractions) was administered on X day to improve airway stenosis (Figures 3A-C). During treatment, the patient's hoarseness and dysphagia improved, and wheezing disappeared. CT scan also showed improvement in airway narrowing and deviation (Figure 1B). Radiotherapy was completed without adverse events on X+14 day, and a CT-guided biopsy of the lung tumor was performed on X+20 day, which showed ACC (Figures 4A-F). The results of Alcian blue and immunohistochemical staining were as follows: positive for Alcian blue, weakly positive for c-kit, positive for gastrointestinal stromal tumor-1, positive for p40, and positive for smooth muscle actin. No lesions were found in the head and neck region. Bronchoscopy showed no lesions in the tracheal mucosa. The patient was then started on systemic therapy with 24 mg of lenvatinib one month after X day. Lenvatinib caused cardiac pain, fever, and myelosuppression (grade 2, Common Terminology Criteria for Adverse Events version 5.0) [7] but was resumed at a lower dose after a break. One month after starting lenvatinib, a CT scan showed a reduction of lung metastases (Figure 2B), but edema around the gallbladder (Figure 2C) and hypothyroidism was observed, and lenvatinib was discontinued. Subsequent chest X-rays showed a re-expansion of the lung metastases. Two months after radiotherapy, there was no evidence of recurrent airway narrowing.

## Discussion

ACC is a malignant tumor that occurs primarily in the head and neck region [1-3]. ACC is classified as a low-grade malignancy and is characterized by relatively slow tumor growth [8], and long-term survival can be expected even in the presence of lung metastases [9]. Despite being a rare disease with no large clinical trials, surgery remains the primary treatment for locally advanced ACC [3]. In comparison to squamous cell carcinoma, ACC in the head and neck region is considered radioresistant. However, it is known for its strong invasiveness, making complete resection difficult, which is why postoperative radiotherapy is considered an important adjunctive treatment [4]. The National Comprehensive Cancer Network guidelines also advocate for postoperative radiotherapy in ACC cases [10]. Reports of conventional X-ray radiotherapy for unresectable head and neck ACC are limited and its efficacy is poor, with five-year local control rates less than 50% [11]. More precise radiotherapies such as intensity-modulated radiation therapy (IMRT) may improve local control rates [12]. Mizoguchi et al. reported a five-year local control rate of 83.1% in 20 patients with head and neck ACC who received definitive radiotherapy (four of whom received IMRT) [13]. They revealed no statistically significant difference in overall survival or local control compared with 24 head and neck ACC patients who received postoperative radiotherapy (nine of whom received IMRT) [13]. In recent years, particle therapy, particularly heavy particle therapy, has been utilized and is considered an effective treatment option with widespread reporting [14,15].

ACC originating from outside the head and neck region, such as the larynx and trachea, is extremely rare [2,5,6], and its treatment has not been established with limited reports on palliative treatment. Kim reported that radiation therapy using 66 Gy in 33 fractions for lung and giant mediastinal tumors resulted in significant shrinkage [5]. They also reported that radiation therapy using 25-35 Gy (2.5 or 3 Gy per fraction) for painful bone metastases provided rapid and lasting pain relief [5].

In the present case, no definitive pathological diagnosis was made at the time of initial radiation therapy, although a mediastinal tumor of thyroid origin was suspected based on imaging results. The patient had multiple lung tumors that were not visible on a chest X-ray taken a year prior and was experiencing respiratory distress due to severe airway constriction. Immediate airway management was deemed necessary due to the rapid progression of the disease, but endotracheal intubation and tracheotomy were not possible. With the patient's consent, radiotherapy was performed as a clinical cancer emergency, while the pathological diagnosis was still uncertain. A dose of 30 Gy in 10 fractions was used, which is a palliative dose for malignant tumors in general. At the start of radiotherapy, the patient was under the policy of best supportive care. However, the patient's condition improved after radiation therapy, and it was determined that systemic therapy was indicated. A pathological diagnosis was then made, leading to the diagnosis of ACC.

## Conclusions

We present a case of an aggressive primary ACC in the mediastinum causing airway obstruction that responded well to palliative radiotherapy. As ACC originating outside the head and neck is rare, conducting extensive clinical trials is challenging. The documentation of such cases will contribute to the development of future diagnostic and treatment strategies.
